# Epidemiological Characteristics and the Development of Prognostic Nomograms of Patients With HIV-Associated Cutaneous T-Cell Lymphoma

**DOI:** 10.3389/fonc.2022.847710

**Published:** 2022-03-15

**Authors:** Zheng Yang, Daoqing Gong, Fei Huang, Yi Sun, Qinming Hu

**Affiliations:** ^1^ Department of Infectious Disease, Jingzhou Hospital, Yangtze University, Jingzhou, China; ^2^ Teaching Office, Jingzhou Hospital, Yangtze University, Jingzhou, China; ^3^ Department of Dermatology, Jingzhou Hosiptal, Yangtze University, Jingzhou, China

**Keywords:** HIV, CTCL, HIV-associated CTCL, SEER, nomogram, prognosis

## Abstract

**Background:**

The incidence of human immunodeficiency virus (HIV) associated cutaneous T-Cell lymphoma (HIV-associated CTCL) is very low, and there is a lack of relevant epidemiological and clinical prognostic studies. Therefore, we aimed to study the epidemiological characteristics of HIV-associated CTCL and to construct and validate a nomogram predicting patient survival.

**Methods:**

Demographic, clinical characteristics, and incidence data from the Surveillance, Epidemiology and End Results (SEER) database were screened for patients with HIV-associated CTCL. Independent prognostic factors in patients with HIV-associated CTCL were analyzed to establish nomograms of overall survival (OS) and disease-specific survival (DSS) rates of patients. The performance of the prediction model was validated by the consistency index (C-index), the area under the receiver operating characteristic curve (AUC), and calibration plots.

**Results:**

A total of 883 eligible patients were screened for inclusion in this study and randomized to the training cohort (70%, n = 619) and the validation cohort (30%, n = 264). The age-adjusted average incidence rate per 100,000 persons per year for HIV-associated CTCL was 0.071 for the period 2004-2017, with an increasing incidence rate. The median age of the included patients was 59 years, of which male Caucasian held a majority. 99.5% of the patients had a tumor tissue subtype of mycosis fungoides, while the other tumor subtypes were sézary syndrome. The median OS for patients with HIV-associated CTCL was 162 months, and the OS rates at 1, 3, 5, and 10 years were 0.964, 0.904, 0.835, and 0.766, respectively. Univariate and multivariate COX regression analysis were performed, and prognostic indicators such as “Age”, “Radiation”, “Chemotherapy”, “Summary stage”, “Sequence number” were ultimately incorporated and used to establish nomograms of OS and DSS rates at 1, 3, 5 and 10 years for the training cohort. The C-index, AUC, and calibration plot confirmed that our prediction model had good accuracy.

**Conclusion:**

While HIV-associated CTCL is very rare, its incidence has been on the rise in the last decade or so. We described the epidemiological characteristics and prognostic factors in patients with HIV-associated CTCL.

## Introduction

People infected with human immunodeficiency virus (HIV) are at a higher risk of developing cancers such as non-Hodgkin’s lymphoma (NHL), Kaposi’s sarcoma, and cervical cancer due to immunosuppression compared to the general population, with a 77-fold increased risk of NHL (1, 2). Although antiretroviral therapy (ART) has reduced mortality among people living with HIV, cancer remains a significant cause of death. The attributable mortality rate of HIV-associated cancers was 386.9 per 100,000 population per year, with NHL causing 3.5% of deaths (3). Among patients with HIV-associated NHL, the three major subtypes, namely, Burkitt lymphoma, diffuse large B-cell lymphoma, and central nervous system lymphoma, exhibit a particularly high risk (4). The incidence of cutaneous T cell lymphoma (CTCL) is 10.2 cases per million people (5), which accounts for 3.4% of all NHL (6). There are very few studies on CTCL in HIV-infected patients.

CTCL is a clinically and biologically heterogeneous group of extranodal NHL characterized by skin involvement, with mycosis fungoides (MF) and sézary syndrome (SS) being the most common tumor subtypes. In the early stage of the onset, patients usually present with discrete skin lesions resembling eczema or extensive erythema. Patients with advanced disease may have fungating tumors or leukemia, eventually involving the lymph nodes and viscera (7). Since the early manifestations of MF/SS are similar to a benign, inflammatory or autoimmune skin disease, it is not easy to detect characteristic histologic changes even after multiple pathologic biopsies. Therefore, the diagnosis of MF/SS requires a comprehensive analysis combining clinical manifestations, pathological assessment and molecular studies of patients, which poses a great challenge to clinical diagnosis. So far, the epidemiological characteristics, clinical features, and long-term prognosis studies of CTCL in HIV-infected patients have rarely been reported. Previously, Wang et al. (8) showed that HIV-infected patients with CTCL had higher survival rates and lower overall risk of death compared with HIV-uninfected patients. However, the epidemiological characteristics of patients with HIV-associated CTCL and the prognostic factors associated with survival remain unclear.

Therefore, this study retrospectively analyzed the epidemiological features, demographic characteristics, and prognostic factors of HIV-associated CTCL based on patient data extracted from the Surveillance, Epidemiology and End Results (SEER) database, and established prognostic nomograms to guide clinicians to more accurately assess the patient’s overall survival (OS) and disease-specific survival (DSS) rates.

## Methods

### Data Source and Patient Selection

**Figure 1:**
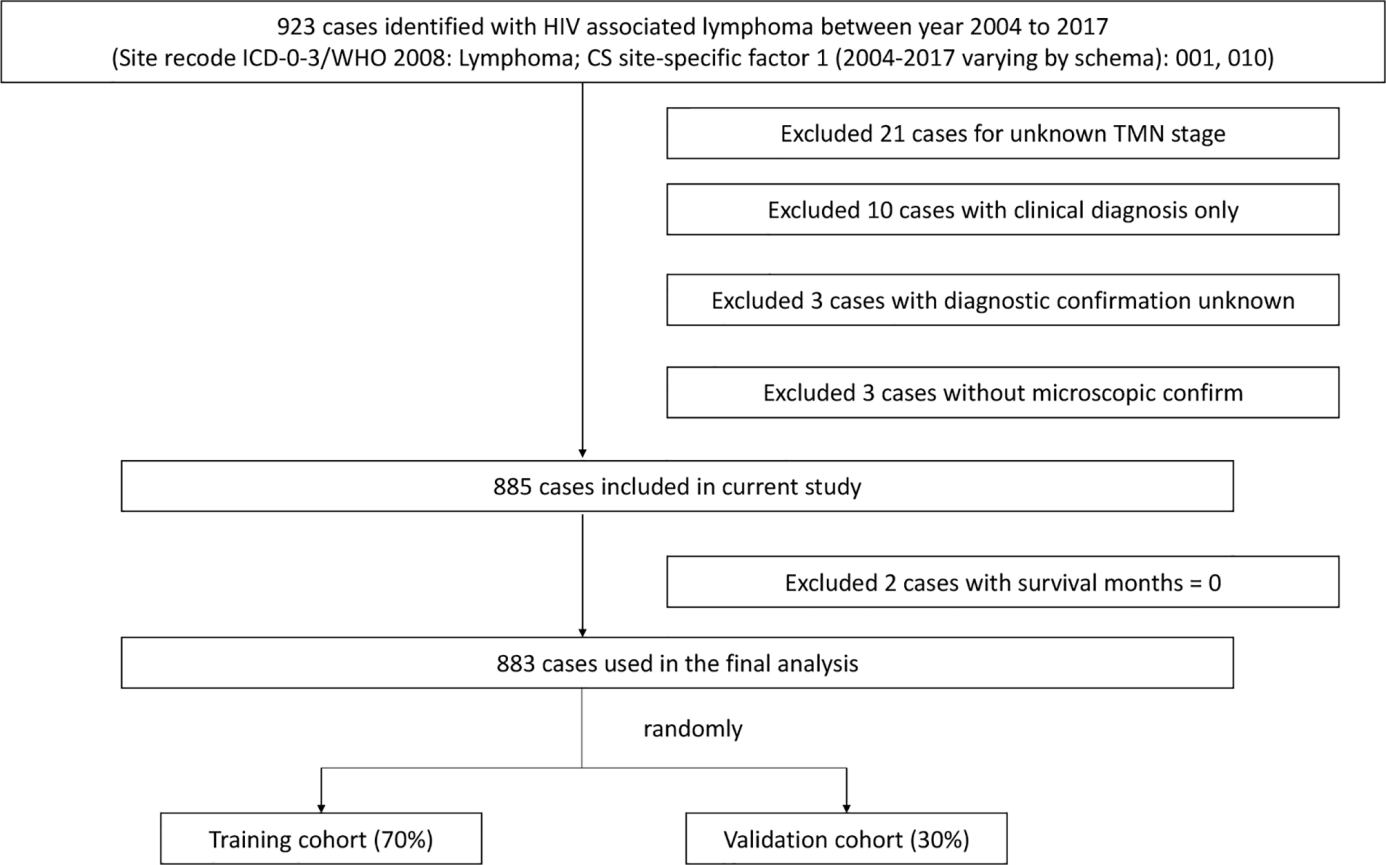
Flowchart of the selection process of patients in the SEER database. SEER, Surveillance, Epidemiology, and End Results.

We used SEER*Stat software (Version 8.3.9.1; National Cancer Institute, Surveillance Research Program) to extract data from patients diagnosed with HIV-associated CTCL from the SEER database [Nov 2020 Sub (2000-2018)] released in April 2021. The SEER database is currently one of the largest publicly available cancer datasets, providing patient data from 18 cancer registries, approximately 28% of all cancer cases in the United States included (9). Institutional ethics committee approval was not required for this study as all patient information was obtained from the online publicly available SEER database with patient anonymity. Patients who meet the following criteria will be included in the analysis: 1. Site recode ICD-0-3/WHO 2008 is lymphoma; 2. CS site-specific factor 1 (2004-2017 varying by schema) is 001 or 010; 3. Diagnosis of lymphoma confirmed by microscopy; 4. Complete tumor stage and diagnostic data; 5. Follow-up survival time ≥1 month. Ultimately, 883 patients with HIV-associated CTCL were included for analysis in this study and randomly divided into two consecutive cohorts: 70% training cohort (n = 619) and 30% validation cohort (n = 264) (**Figure 1**).

### Variable Selection

Relevant variables of interest for all patients were extracted from the SEER database including year of diagnosis, race, marital status, gender, age, primary site, surgery, radiotherapy, chemotherapy, tumor stage, number of primary tumors, histologic subtype, survival time, vital status, cause of death, and outcome variables: OS and DSS. OS refers to the time from the diagnosis of HIV-associated CTCL to the death of the patient from any cause, DSS the time from the diagnosis of HIV-associated CTCL to the death of the patient due to the cause.

### Statistical Analysis

The annual incidence of HIV-associated CTCL per 100,000 population was calculated by SEER*Stat software, adjusted for the age of the standard US population in 2000. To analyze trends in incidence rates, we also used the Joinpoint Regression Program 4.9.0.0 -March, 2021 software provided by National Cancer Institute (NCI) to calculate annual percentage change (APC).

The demographic characteristics, Cox regression analysis, Kaplan-Meier survival analysis, and the drawing of nomograms, ROC curves, and calibration plots in this study were all run using R software [R version 4.1.1 (2021-08-10); http://www.r-project.org]. X-tile software (version 3.6.1) was used to classify patients into low, medium and high risk and to perform statistical analysis. All reported statistical significance tests were two-tailed, and P < 0.05 was considered statistically significant. The main R packages applied in this study are foreign, caret, compareGroups, HardyWeinberg, survminer, survival, rms, survivalROC, etc. Included patients were randomized into 70% training cohort and 30% validation cohort based on the caret R package. Hardy Weinberg, compareGroups R package was used for demographic analysis, in which categorical variables were expressed as percentages (%). The survminer, survival R package was applied to Kaplan-Meier survival analysis. We used the survival, rms R package to plot nomograms and calibration curves, as well as to perform Cox regression; survivalROC was used to plot ROC curves.

## Results

### Demographic and Clinical Characteristics

Based on the inclusion criteria, this study finally obtained the data of 883 patients diagnosed from 2004 to 2017 from the SEER database, and then randomly divided them into two consecutive cohorts: the training cohort and the validation cohort (**Figure 1**). From **Table 1**, we found no statistical differences in the aspect of demographic and clinical characteristics between two cohorts. In this study, 60% of patients were male and the majority were white (73.0%), followed by black (16.5%). The median age of patients was 59 years [95% confidence interval (CI), 49-69], with a minimum age of 4 years and a maximum age of 85 years, and 53.0% of patients were <60 years old. The primary site of tumors in the included patients was all from the skin, which were HIV-associated CTCL. The tumor tissue subtypes of HIV-associated CTCL were mycosis fungoides (99.5%) and sézary syndrome (0.45%). According to the summary staging system, the patients with localized staging held the majority (79.0%), and the proportion of patients with regional, distant, and unknown/unstaged staging was 15.7%, 1.70%, and 3.51%, respectively. The main treatment options were surgery, radiotherapy, and chemotherapy, with 19.6% of patients undergoing surgery, 23.1% receiving chemotherapy, and 10.2% receiving radiotherapy. 23.7% of patients were found to have more than one primary tumor at follow-up (**Table 1**).

**Table 1:** Demographic and clinical characteristics of patients with HIV-associated CTCL.

Characteristic	Total cohort	Training cohort	Validation cohort	*P* value
	(*n=*883)	(*n*=619)	(*n*=264)	
**Year of diagnosis**				0.224
2004-2008	31 (3.51%)	26 (4.20%)	5 (1.89%)	
2009-2013	332 (37.6%)	233 (37.6%)	99 (37.5%)	
2014-2017	520 (58.9%)	360 (58.2%)	160 (60.6%)	
**Sex**				0.371
Female	353 (40.0%)	241 (38.9%)	112 (42.4%)	
Male	530 (60.0%)	378 (61.1%)	152 (57.6%)	
**Race**				0.355
White	645 (73.0%)	449 (72.5%)	196 (74.2%)	
Black	146 (16.5%)	101 (16.3%)	45 (17.0%)	
Asian or Pacific Islander	74 (8.38%)	58 (9.37%)	16 (6.06%)	
Other	18 (2.04%)	11 (1.78%)	7 (2.65%)	
**Age**				0.646
<60	468 (53.0%)	331 (53.5%)	137 (51.9%)	
60-69	211 (23.9%)	145 (23.4%)	66 (25.0%)	
70-79	135 (15.3%)	91 (14.7%)	44 (16.7%)	
≥80	69 (7.81%)	52 (8.40%)	17 (6.44%)	
**Marital status**				0.969
Married	437 (49.5%)	307 (49.6%)	130 (49.2%)	
Unmarried	296 (33.5%)	206 (33.3%)	90 (34.1%)	
Unknown	150 (17.0%)	106 (17.1%)	44 (16.7%)	
**Primary Site**				0.350
Skin, NOS	507 (57.4%)	348 (56.2%)	159 (60.2%)	
Skin of the extremities	157 (17.8%)	117 (18.9%)	40 (15.2%)	
Skin of trunk	150 (17.0%)	106 (17.1%)	44 (16.7%)	
Skin of the head and face	36 (4.08%)	28 (4.52%)	8 (3.03%)	
Others	33 (3.74%)	20 (3.23%)	13 (4.92%)	
**Surgery**				0.607
No	710 (80.4%)	501 (80.9%)	209 (79.2%)	
Yes	173 (19.6%)	118 (19.1%)	55 (20.8%)	
**Radiation**				0.408
No/Unknown	793 (89.8%)	552 (89.2%)	241 (91.3%)	
Yes	90 (10.2%)	67 (10.8%)	23 (8.71%)	
**Chemotherapy**				0.258
No/Unknown	679 (76.9%)	469 (75.8%)	210 (79.5%)	
Yes	204 (23.1%)	150 (24.2%)	54 (20.5%)	
**Summary stage**				0.575
Localized	698 (79.0%)	489 (79.0%)	209 (79.2%)	
Regional	139 (15.7%)	96 (15.5%)	43 (16.3%)	
Distant	15 (1.70%)	13 (2.10%)	2 (0.76%)	
Unknown/unstaged	31 (3.51%)	21 (3.39%)	10 (3.79%)	
**T stage**				0.695
T1	503 (57.0%)	358 (57.8%)	145 (54.9%)	
T2	214 (24.2%)	142 (22.9%)	72 (27.3%)	
T3	79 (8.95%)	55 (8.89%)	24 (9.09%)	
T4	56 (6.34%)	41 (6.62%)	15 (5.68%)	
TX	31 (3.51%)	23 (3.72%)	8 (3.03%)	
**N stage**				0.304
N0	813 (92.1%)	568 (91.8%)	245 (92.8%)	
N1	37 (4.19%)	30 (4.85%)	7 (2.65%)	
N2	4 (0.45%)	3 (0.48%)	1 (0.38%)	
N3	5 (0.57%)	2 (0.32%)	3 (1.14%)	
NX	24 (2.72%)	16 (2.58%)	8 (3.03%)	
**M stage**				0.280
M0	866 (98.1%)	604 (97.6%)	262 (99.2%)	
M1	13 (1.47%)	11 (1.78%)	2 (0.76%)	
MX	4 (0.45%)	4 (0.65%)	0 (0.00%)	
**Stage**				0.945
I	689 (78.0%)	480 (77.5%)	209 (79.2%)	
II	85 (9.63%)	62 (10.0%)	23 (8.71%)	
III	44 (4.98%)	32 (5.17%)	12 (4.55%)	
IV	22 (2.49%)	16 (2.58%)	6 (2.27%)	
unknown	43 (4.87%)	29 (4.68%)	14 (5.30%)	
**Sequence number**				0.298
Only 1 primary tumor	674 (76.3%)	479 (77.4%)	195 (73.9%)	
More than 1 primary tumors	209 (23.7%)	140 (22.6%)	69 (26.1%)	
**Histologic subtype**				0.323
Mycosis fungoides	879 (99.5%)	615 (99.4%)	264 (100%)	
Sezary syndrome	4 (0.45%)	4 (0.65%)	0 (0.00%)	

NOS, not otherwise specified; HIV-associated CTCL, human immunodeficiency virus associated cutaneous T-Cell lymphoma.

### Incidence of HIV-Associated CTCL

**Figure 2:**
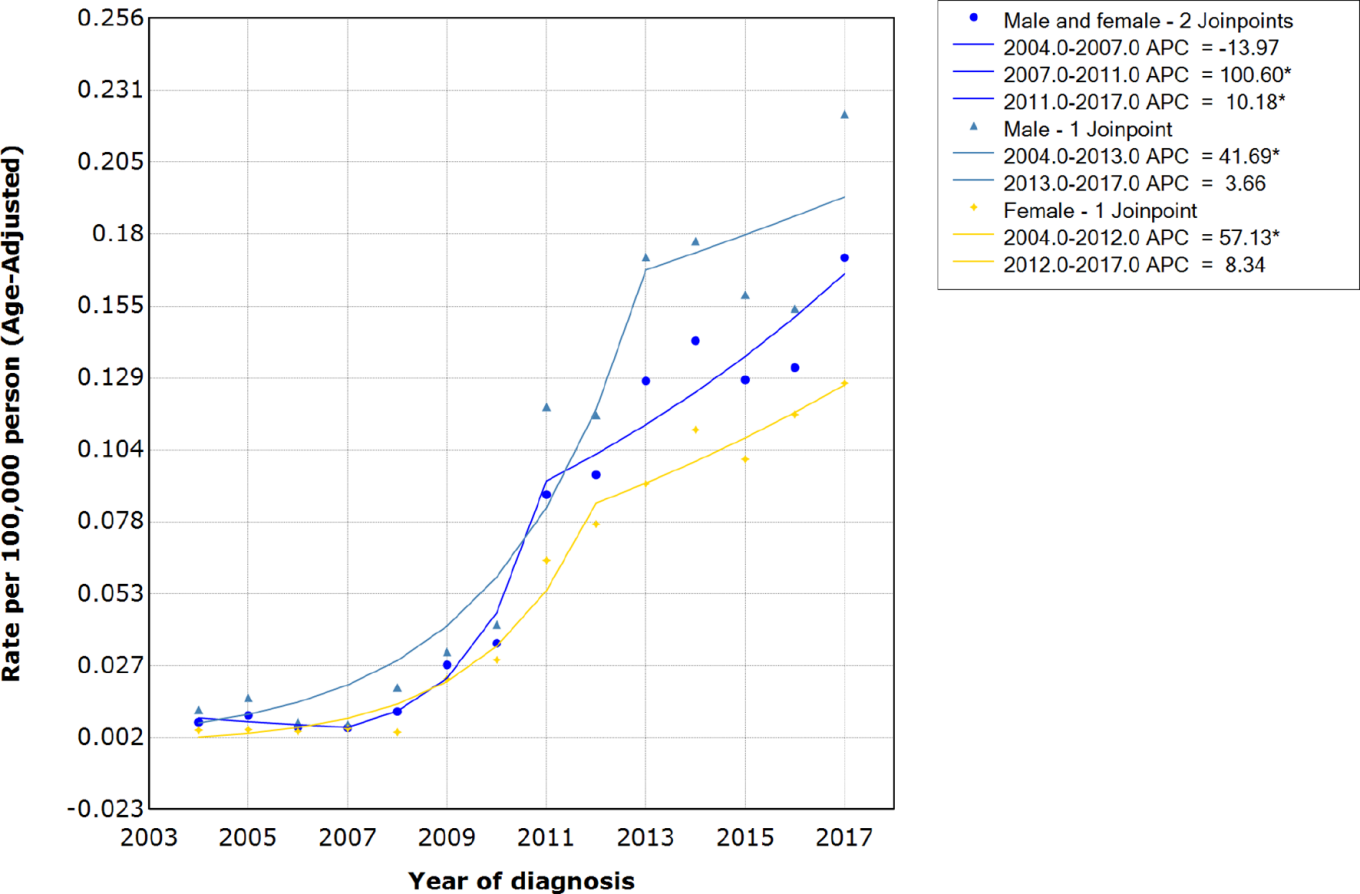
Incidence rates and APCs of patients with HIV-associated CTCL. APCs, annual percentage changes; HIV-associated CTCL, human immunodeficiency virus associated cutaneous T-Cell lymphoma.

The age-adjusted average incidence rate per 100,000 persons per year for HIV-associated CTCL was 0.071 during the period 2004-2017. The incidence of HIV-associated CTCL remained stable from 2004 to 2007, with an average annual incidence of 0.01/100,000, and an APC of -13.97 (95% CI, -65.3-113.4; P=0.699). However, since 2007, the annual incidence of HIV-associated CTCL per 100,000 people increased sharply from 0.01 to 0.09 in 2011, with an APC of 100.60 (95% CI, 19.9-235.5; P= 0.016). The increase in incidence rate per 100,000 person-years leveled off between 2011-2017, increasing from 0.09 in 2011 to 0.17 in 2017, with an APC of 10.18 (95% CI, 3.2-17.7; P= 0.011) (see **Figure 2** and **Supplementary Tables 1**, **2**).

### Survival Analysis

**Figure 3:**
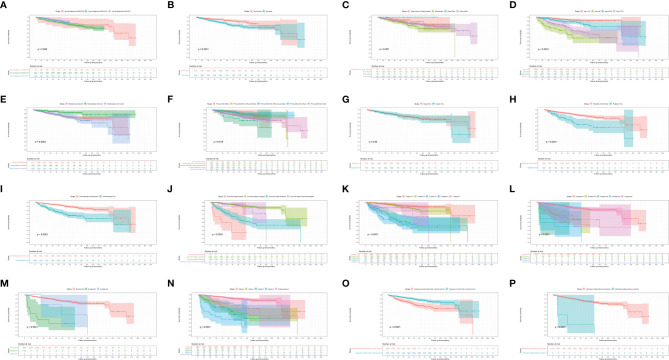
Kaplan-Meier survival analysis of the effect of each independent predictor on OS in patients with HIV-associated CTCL. **(A–P)** The survival curves of year of diagnosis, sex, race, age, marital status, primary site, surgery, radiation, chemotherapy, summary stage, t stage, n stage, m stage, stage, sequence number, histologic subtype, respectively. OS, overall survival; HIV-associated CTCL, human immunodeficiency virus associated cutaneous T-Cell lymphoma.

**Figure 4:**
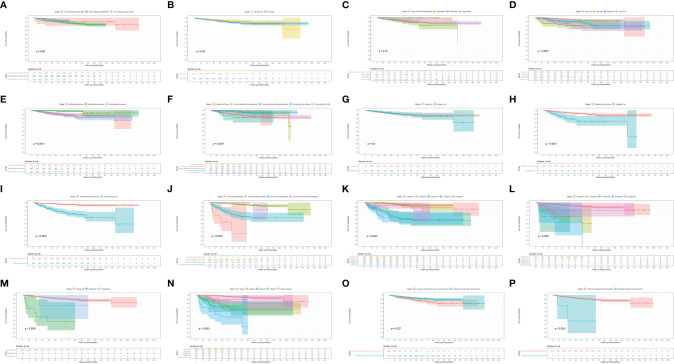
Kaplan-Meier survival analysis of the effect of each independent predictor on DSS in patients with HIV-associated CTCL. **(A–P)** The survival curves of year of diagnosis, sex, race, age, marital status, primary site, surgery, radiation, chemotherapy, summary stage, t stage, n stage, m stage, stage, sequence number, histologic subtype, respectively. DSS, disease-specific survival; HIV-associated CTCL, human immunodeficiency virus associated cutaneous T-Cell lymphoma.

**Figure 5:**
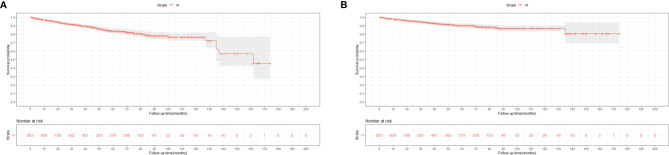
Kaplan-Meier survival analysis of all patients with HIV-associated CTCL: **(A)** OS; **(B).** DSS. HIV-associated CTCL, human immunodeficiency virus associated cutaneous T-Cell. lymphoma; OS, overall survival; DSS, disease-specific survival.

**Figures 3**, **4** show the survival curves of OS, DSS for all patients with HIV-associated CTCL in each univariate condition, respectively. Kaplan-Meier survival analysis showed that OS and DSS decreased with increasing patient age, and the OS of women was longer than that of men. In terms of treatment options, chemotherapy and radiotherapy were associated with OS and DSS. Interestingly, chemotherapy and radiotherapy significantly reduced patient OS and DSS (P < 0.0001), whereas surgery had no statistically significant effect on OS and DSS. In addition, marital status, tumor primary site, tumor stage, tumor tissue subtype, and number of primary tumors were all associated with OS and DSS. Race and the year of diagnosis had no effect on the OS and DSS. **Figure 5** shows the survival curves of OS, DSS for all patients with HIV-associated CTCL. The median OS for all patients was 162 months, and their OS rates at 1, 3, 5, and 10 years were 0.964 (95% CI: 0.952- 0.977), 0.904 (95% CI: 0.883-0.925), 0.835 (95% CI: 0.805-0.866), and 0.766 (95% CI: 0.716- 0.819). We also evaluated the OS and DSS rates of HIV-associated CTCL patients at 1, 3, and 5 years using different tumor staging systems (**Table 3**).

### Univariate, Multivariate COX Regression Analysis and Nomogram

The results of univariate and multivariate Cox regression analyses of OS and DSS in HIV-associated CTCL patients are shown in **Table 2**. Univariate Cox regression analysis showed that the variables “Sex”, “Age”, “Radiation”, “Chemotherapy”, “Summary stage”, “Stage”, “T stage”, “N stage”, “M stage’, “Sequence number”, and “Histologic subtype” were associated with patient OS. It was found that “Age”, “Radiation”, “Chemotherapy”, “Sequence number” were associated with patient OS (all P<0.05), after the above univariate results were included in the multivariate COX regression analysis. Similarly, univariate Cox regression analysis showed that variables such as “Age”, “Radiation”, “Chemotherapy”, “Summary stage”, and “Histologic subtype” were associated with DSS. Analysis of these univariate variables included in multivariate COX regression showed that “Age”, “Chemotherapy”, and “Summary stage” were associated with DSS (all *P* < 0.05).

**Table 2:** Univariate and multivariate Cox regression analysis of OS and DSS in patients with HIV-associated CTCL in training cohort.

Characteristics	OS						DSS					
	Univariate analysis			Multivariate analysis			Univariate analysis			Multivariate analysis		
	HR	95% CI	p Value	HR	95% CI	p Value	HR	95% CI	p Value	HR	95% CI	p Value
**Year of diagnosis**												
2004-2008	Reference						Reference					
2009-2013	1.038	0.439-2.455	0.933				1.304	0.399-4.269	0.661			
2014-2017	0.693	0.279-1.719	0.429				0.692	0.199-2.410	0.563			
**Sex**												
Female	Reference			Reference			Reference					
Male	1.957	1.218-3.145	0.006	1.663	0.998-2.772	0.051	1.338	0.760-2.357	0.313			
**Race**												
Asian or Pacific Islander	Reference						Reference					
White	2.036	0.741-5.591	0.168				2.507	0.605-10.380	0.205			
Black	2.608	0.882-7.711	0.083				3.493	0.781-15.620	0.102			
Other	0.000	0.000- Inf	0.995				0.000	0.000-Inf	0.996			
**Age**												
<60	Reference			Reference			Reference			Reference		
60-69	2.392	1.286-4.450	0.006	2.298	1.191-4.433	**0.013**	2.237	1.105-4.527	0.025	2.429	1.191-4.954	**0.015**
70-79	5.261	2.974-9.307	<0.001	4.227	2.242-7.967	**<0.001**	3.257	1.590-6.675	<0.010	3.101	1.477-6.508	**<0.010**
≥80	9.487	5.125-17.560	<0.001	7.854	3.890-15.856	**<0.001**	4.824	2.127-10.941	<0.001	4.828	2.021-11.530	**<0.001**
**Marital status**												
Married	Reference						Reference					
Unmarried	1.208	0.777-1.877	0.401				1.382	0.796-2.398	0.250			
Unknown	0.444	0.210-0.939	0.034				0.293	0.089-0.965	0.044			
**Primary Site**												
Others	Reference						Reference					
Skin, NOS	1.290	0.404-4.123	0.667				37293683.000	0- Inf	0.996			
Skin of trunk	0.486	0.126-1.883	0.297				8929581.000	0- Inf	0.997			
Skin of the head and face	2.073	0.536-8.024	0.291				60899953.000	0- Inf	0.996			
Skin of the extremities	0.839	0.238-2.949	0.784				16428213.000	0- Inf	0.996			
**Surgery**												
No	Reference						Reference					
Yes	0.983	0.590-1.639	0.949				1.229	0.656-2.301	0.520			
**Radiation**												
No/Unknown	Reference						Reference			Reference		
Yes	2.470	1.486-4.105	<0.001	1.915	1.050-3.493	**0.034**	3.509	1.932-6.373	<0.001	1.885	0.982-3.616	0.057
**Chemotherapy**												
No/Unknown	Reference						Reference			Reference		
Yes	2.584	1.703-3.918	<0.001	2.128	1.335-3.393	**0.002**	5.145	2.976-8.896	<0.001	3.321	1.854- 5.951	**<0.001**
**Summary stage**												
Distant	Reference			Reference			Reference			Reference		
Localized	0.067	0.030-0.151	<0.001	0.367	0.029-4.588	0.437	0.040	0.016-0.100	<0.001	0.066	0.024-0.180	**<0.001**
Regional	0.341	0.150-0.776	<0.05	0.393	0.045-3.407	0.397	0.311	0.127-0.766	0.011	0.333	0.130-0.852	**0.022**
Unknown/unstaged	0.176	0.051-0.607	<0.01	0.298	0.016-5.403	0.412	0.051	0.006-0.431	0.006	0.097	0.010-0.902	**0.040**
**T stage**												
T1	Reference						Reference					
T2	1.352	0.716-2.553	0.353	1.421	0.738-2.736	0.294	1.108	0.430-2.856	0.832			
T3	5.399	3.049-9.560	<0.001	2.298	0.424-12.463	0.335	9.128	4.499-18.519	<0.001			
T4	7.690	4.318-13.696	<0.001	3.357	0.486-23.208	0.220	11.210	5.398-23.279	<0.001			
TX	4.530	1.986-10.334	<0.001	4.311	0.443-41.978	0.208	3.682	1.065-12.732	0.040			
**N stage**												
N0	Reference						Reference					
N1	3.884	2.049-7.360	<0.001	1.621	0.651-4.040	0.300	5.099	2.473-10.516	<0.001			
N2	12.933	3.152-53.062	<0.001	40.619	4.769-345.958	<0.001	10.225	1.399-74.731	0.022			
N3	10.207	1.406- 74.080	<0.05	22.056	0.650-748.619	0.085	17.175	2.334-126.387	<0.010			
NX	3.269	1.407-7.594	<0.01	1.482	0.339-6.4708	0.601	1.960	0.472-8.138	0.354			
**M stage**												
M0	Reference						Reference					
M1	11.221	5.384-23.390	<0.001	6.483	0.328-128.178	0.220	13.274	5.635-31.260	<0.001			
MX	4.501	1.415-14.320	0.011	1.573	0.278-8.897	0.608	2.456	0.338-17.850	0.375			
**Stage**												
I	Reference						Reference					
II	4.195	2.426-7.254	<0.001	1.263	0.201-7.925	0.803	6.802	3.408-13.580	<0.001			
III	6.082	3.248-11.389	<0.001	0.956	0.096-9.570	0.970	10.200	4.728-22.007	<0.001			
IV	14.009	6.975-28.138	<0.001	0.181	0.009-3.852	0.273	22.855	9.943-52.536	<0.001			
unknown	3.011	1.348-6.725	0.007	0.607	0.060-6.147	0.673	2.809	0.830-9.506	0.097			
**Sequence number**												
More than 1 primary tumors	Reference						Reference					
Only 1 primary tumor	0.411	0.270-0.625	<0.001	0.591	0.363-0.963	**0.035**	0.589	0.334-1.037	0.067			
**Histologic subtype**												
Mycosis fungoides	Reference						Reference			Reference		
Sezary syndrome	11.700	4.275-32.020	<0.001	0.911	0.158-5.236	0.916	9.140	2.218-37.670	0.002	1.319	0.290-6.007	0.720

CI, confidence intervals; HR, hazard ratio; Inf, infinity; OS, overall survival; DSS, disease-specific survival; HIV-associated CTCL, human immunodeficiency virus associated cutaneous T-Cell lymphoma. The values in bold highlight p-values <0.05.

**Figure 6:**
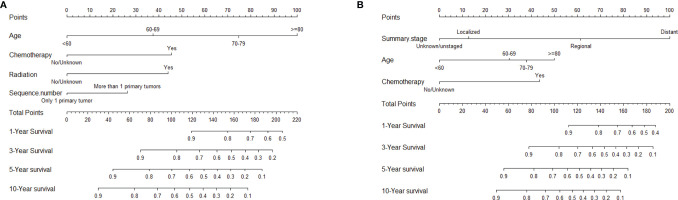
Nomograms to predict 1-, 3-, 5-, and 10-year OS and DSS for patients with HIV-associated CTCL: **(A)** OS; **(B)** DSS. HIV-associated CTCL, human immunodeficiency virus associated cutaneous T-Cell lymphoma; OS, overall survival; DSS, disease-specific survival.

The nomogram, based on characteristic phenotype of disease, is used to predict the future probability of a specific outcome event in a population with a specific characteristic. After excluding non-significant variables, the statistically significant variables in the multivariate COX regression were used to construct the nomogram of OS and DSS in the training cohort. Each variable corresponds to a specific point, through drawing a straight line on the point axis, and then the sum of the values corresponding to these points can predict the probability of OS and DSS in 1, 3, 5 and 10 years (**Figures 6A, B** **)**.

### Nomogram Verification

**Figure 7:**
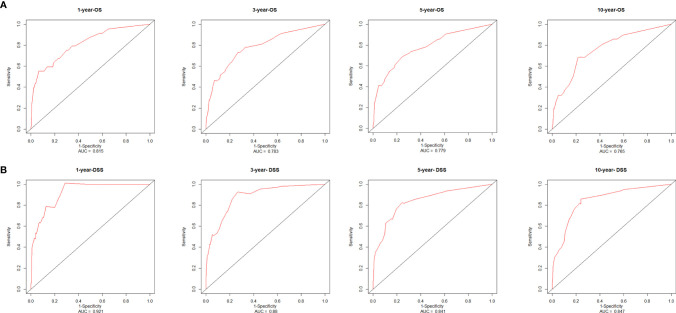
Receiver operating characteristic curves and area under the curve values from the nomograms for 1-, 3-, 5-, and 10-year OS **(A)** and DSS **(B)**. AUC, area under the receiver operating characteristic curve; OS, overall survival; DSS, disease-specific survival.

**Figure 8:**
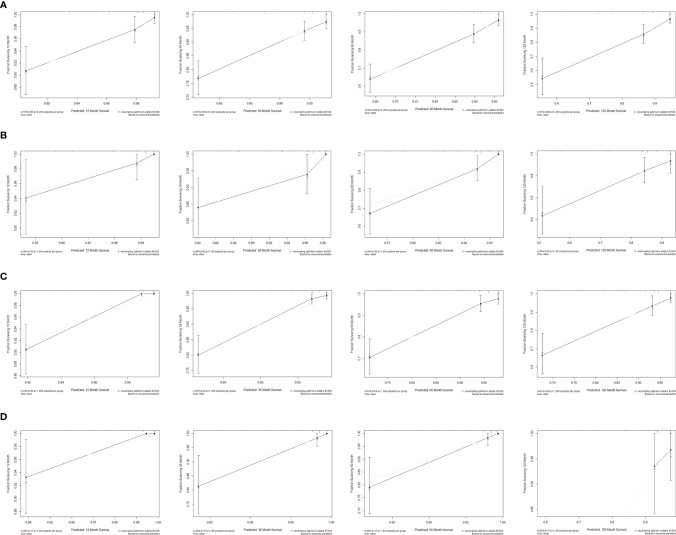
Calibration plots of the nomograms for predicting 1-, 3-, 5-, and 10-year OS and DSS using the training and validation cohort. **(A)** 1-, 3-, 5-, and 10-year calibration plots of OS using training cohort; **(B)** 1-, 3-, 5-, and 10-year calibration plots of OS using validation cohort; **(C)** 1-, 3-, 5-, and 10-year calibration plots of DSS using training cohort; **(D)** 1-, 3-, 5-, and 10-year calibration plots of DSS using validation cohort. OS, overall survival; DSS, disease-specific survival.

The consistency index (C-index) and the area under the receiver operating characteristic curve (AUC) were used to assess the discrimination of the nomogram. The C-index of the nomogram, which was used to predict OS in this study, was 0.78 in the training cohort. AUCs were plotted to verify the accuracy of the nomogram to predict the OS at 1, 3, 5 and 10 years. The results showed that the AUCs were 0.815, 0.783, 0.779 and 0.765 respectively, which indicated good discrimination of the nomogram of OS (**Figure 7A**). The nomogram of DSS was better distinguished with a C-index of 0.848, and AUCs of 1-, 3-, 5-, and 10-year DSS were 0.921, 0.880, 0.841, and 0.847, respectively (**Figure 7B**). The calibration plots were drawn to evaluate the consistency of the nomograms. In this study, the calibration plots showed excellent agreement between the predicted and actual results of OS and DSS in the training cohort and the validation cohort (**Figure 8**).

### The Performance of Nomogram in Risk Stratification

**Figure 9:**
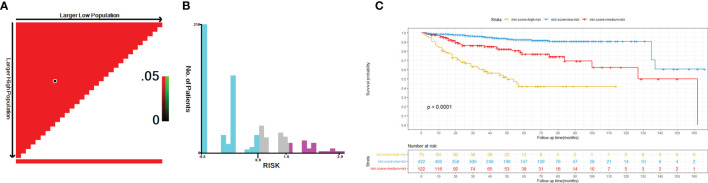
Optimal cutoff values of risk score identified by X-tile analysis of patients with HIV-associated CTCL in the training cohort **(A, B)**. Overall survival of patients with HIV-associated CTCL stratified by risk score **(C)**. HIV-associated CTCL, human immunodeficiency virus associated cutaneous T-Cell lymphoma.

To further assess the discrimination of the nomogram, we first classified the 619 patients in the training cohort into 422 low-risk, 122 medium-risk, and 75 high-risk populations using X-tile software, with cutoff values of 0.92 and 1.67 as shown in **Figures 9A, B**. Then, the results of Kaplan-Meier survival analysis showed that the low-risk population had the best OS, followed by the medium-risk population, and the high-risk population the worst (*P* < 0.0001) (**Figure 9C**).

## Discussion

CTCL belongs to a relatively rare group of NHL, a heterogeneous group of lymphoproliferative diseases characterized by clonal proliferation of mature post-thymic T cells infiltrating the skin (10). At present, the detailed pathogenesis of CTCL remains largely unknown, and known risk factors include age, race, gender, and environmental, infectious, iatrogenic, and other exposures associated with the occurrence of CTCL. Among these, infectious factors including human T lymphotropic virus type-1 (HTLV-1), HTLV-2, Epstein-Barr virus (EBV), cytomegalovirus (CMV), human herpesvirus, merkel cell polyoma virus (MCV), and HIV are also considered to play an important role in pathogenesis (11). The global incidence of CTCL has increased 2 to 3 times in the last two decades, with 5 to 11 cases per 1,000,000 people per year (6, 12). The incidence of CTCL varies according to geographical location (11).

However, CTCL is uncommonly associated with HIV infection, and the incidence of HIV-associated CTCL is currently unclear (13). We calculated the incidence of HIV-associated CTCL by extracting patient information from the SEER database. To our knowledge, this is the largest study of HIV-associated CTCL to date.

Our study showed that the age-adjusted average incidence rate of HIV-associated CTCL per 100,000 persons per year for the period 2004-2017 was 0.071. The incidence of HIV-associated CTCL remained stable from 2004 to 2007, with an average annual incidence of 0.01/100,000. However, since 2007, the incidence rate of HIV-associated CTCL had increased sharply to 0.09 per 100,000 person-years by 2011. The incidence rate of HIV-associated CTCL per 100,000 person-years increased from 0.09 in 2011 to 0.17 in 2017.

CTCL can occur at any age, but more often the median age at diagnosis is 54 years (range, 10 - 89 years) with a male-to-female ratio of 1.6:1 (14), and our findings are similar in the HIV-associated CTCL population, with a median age at diagnosis of 59 years and a male-to-female ratio of 1.5:1. Our study showed that OS and DSS decreased with increasing age in HIV-associated CTCL patients. Similarly, another study showed that age >60 years predicted lower survival in CTCL patients (15). There is now growing evidence that the incidence of cancer increases with age, which may be related to various age-related changes such as immune system disorders and DNA damage (16, 17). In addition, the incidence of complications such as cardiovascular, renal, neurocognitive, and osteoporosis increases with age in HIV-infected patients (18). More comorbidities in patients may also have a direct adverse effect on the survival time of patients, which is the cause of the higher mortality rate in elderly patients (19).

In this study, female patients had longer OS than male patients, which may be related to the direct impact of estrogen on the proliferation of lymphoid cells or its effect on anti-tumor immune response (20). Treatment of NHL is dominated by combination chemotherapy regimens, and in female patients, the combination of rituximab can prolong overall survival. Better outcomes in terms of complete remission in certain lymphoid malignancies were associated with a significantly lower clearance of rituximab in female patients than in male patients (21). Marital status is also considered an independent prognostic factor for survival in various cancers, and it has been shown that marital status is a good predictor of prognosis in patients with HL, with widowed patients likely to have worse survival outcomes than other groups (22). Another study, consistent with our findings, showed that NHL patients who lacked partner or spousal support or had a high disease burden may severely impair health-related quality of life (23).

CTCL patients are at a significantly increased risk of developing a second primary malignancy, particularly lymphoma, and this risk increases over time (24). The vast majority of CTCL patients consists of MF subtypes (25, 26). Studies have shown that CTCL is more prevalent in Asian populations, with the MF subtype accounting for 62% of CTCL compared to 3% for the SS subtype (27). And in our study, MF subtypes accounted for 99.5% of HIV-associated CTCL patients. Patients with MF may have underlying immune dysregulation, including T-cell immunodeficiency resulting in reduced immune surveillance in the MF environment, which may be a factor in increasing the risk of malignancy (28), and the same immunodeficiency occurs in HIV-infected patients. Patients with MF have a significantly increased risk of being diagnosed with second primary malignancies such as NHL, HL, melanoma, lung cancer, female breast cancer, prostate cancer, colon cancer, and kidney cancer. OS was lower in MF patients with a second malignancy compared with patients without second malignancies (29). As shown in **Figures 3**, **4**, similar results also appeared in the HIV-associated CTCL population.

In 2007, the International Society for Cutaneous Lymphoma (ISCL) and the European Organization for Research and Treatment of Cancer (EORTC) revised the classification and staging system of CTCL into a TNMB staging system specifically for MF/SS (30), and recommended its use in defining the burden of disease to guide physicians in their treatment strategies. A retrospective study by Agar et al. (14) validated the revised ISCL/EORTC MF/SS staging recommendations and confirmed that the revised T, N, M and B classifications were significantly associated with OS and DSS. As shown in **Table 3**, the tumor staging system in this study included Summary stage, T N M stage, Stage, etc., and to our knowledge, this would be the first study to assess the prognosis of patients with HIV-associated CTCL using a tumor staging system. Numerous studies in the past have shown that the prognosis of patients with HIV-associated NHL in the post-antiretroviral therapy era is significantly better than before (31–34). The prognosis for HIV-positive NHL patients is now very close to or similar to that of HIV-negative NHL patients (35). Prognostic studies in patients with HIV-associated CTCL appear to have better results. Compared with the results of Agar et al. (14), the 5-year OS and DSS of HIV-associated CTCL patients were higher, which is consistent with the results of Wang et al. (8) showing that HIV infection status in CTCL patients is an independent protective factor.

**Table 3:** Summary of OS and DSS rates at years 1, 3, and 5 in patients with HIV-associated CTCL under different tumor staging systems.

	1-year		3-year		5-year	
	OS (%)	DSS (%)	OS (%)	DSS (%)	OS (%)	DSS (%)
**Stage**						
I	99.00	99.50	95.60	98.00	90.44	95.35
II	89.32	90.47	76.80	80.13	60.18	71.20
III	88.52	88.52	69.01	71.77	51.70	65.79
IV	59.10	71.00	45.00	54.10	39.40	47.30
**Summary stage**						
Localized	99.00	99.60	95.30	97.70	90.32	95.11
Regional	86.18	88.25	68.31	74.11	53.40	66.61
Distant	65.00	70.00	49.50	53.30	24.80	26.67
**T stage**						
T1	99.00	99.20	94.95	97.20	90.89	95.80
T2	98.60	100.00	95.37	98.47	87.33	92.85
T3	87.24	88.47	71.80	75.47	52.60	62.39
T4	81.99	85.18	57.88	65.03	45.50	60.39
**N stage**						
N0	97.90	98.40	93.00	95.50	NR	NR
N1	81.08	83.70	62.13	66.51	NR	NR
N2	50.00	66.70	50.00	66.70	NR	NR
N3	60.00	80.00	40.00	53.30	NR	NR
**M stage**						
M0	97.10	97.70	91.30	94.30	84.74	90.98
M1	53.80	67.10	38.50	48.00	28.80	36.00

NR, not reached; OS, overall survival; DSS, disease-specific survival; HIV-associated CTCL, human immunodeficiency virus associated cutaneous T-Cell lymphoma.

Since the introduction of highly active antiretroviral therapy (HAART) in 1996, the life expectancy of HIV-infected patients has increased significantly, thereby increasing the likelihood of cancer in these patients (36). Studies have shown that despite HAART is used in HIV/AIDS patients in nearly 10 years, the survival rate of patients with HIV-related lymphoma is still poor (37). In this study, chemotherapy and radiotherapy were related with OS and DSS in patients with HIV-associated CTCL, but significantly decreased OS and DSS in patients, which was different from previously reported results. The prognosis of patients with HIV infection-associated tumors after chemotherapy and/or radiotherapy is related to their immune status, and lower CD4 counts in patients after treatment are associated with an increased risk of death (38). Another study showed that HIV patients with a CD4 count <200 cells/μL before treatment had an increased likelihood of toxicity after chemotherapy and radiation therapy, which may affect patient survival (39). Due to the lack of patient CD4 count information in this study, and the patient’s specific chemotherapy and radiotherapy regimens cannot be obtained from the SEER database, the results of this study may be biased. Mehta-Shah et al. recommended that patients with early CTCL disease and skin involvement use skin-directed therapies (such as local therapy, phototherapy, radiation therapy, total skin electron beam therapy) without major cumulative toxicity, while systemic therapy (brentuximab vedotin, romidepsin, bexarotene, pralatrexate, vorinostat, methotrexate, mogamulizumab, alemtuzumab, and pembrolizumab) be required for patients with advanced disease. Systemic treatments can be combined with skin directed treatments to provide maximum cumulative efficacy without cumulative toxicity (40). Recent studies have revealed that ectopic expression of paternally expressed gene 10 (PEG10) in large cell transformation (LCT) malignant T cells and suggested that PEG10 inhibition may serve as a promising therapeutic approach for patients with advanced CTCL (41). Nowadays, for patients with CTCL, allogeneic hematopoietic stem cell transplantation has been the only curative treatment (42). However, there is currently no large-sample randomized controlled trial to verify the specific treatment options for patients with HIV-associated CTCL, and further research on related treatment strategies is needed.

Nomogram converts scores for several independent variables into visual graphs, making the results of predictive models more readable and facilitating the assessment of individualized patient risk (43). The nomogram is increasingly being used for prognostic assessment of cancer patients (44). In this study, based on the results of univariate and multivariate COX regression analysis, we finally included “Age”, “Radiation”, “Chemotherapy”, “Summary stage”, “Sequence number” indicators to establish the nomogram of OS and DSS of patients. The C-index of the nomograms used to predict the OS and DSS of patients in the training cohort was 0.78 and 0.848, respectively, indicating that the model has good discrimination. The calibration plot also showed the consistency between the predicted and actual results of OS and DSS in two cohorts. All of these results suggested that the nomograms we constructed had good clinical predictive value.

Nevertheless, there exist some limitations in this study. Firstly, this study was conducted by retrospectively analyzing the information in the SEER database, thus making it impossible to guarantee the completeness and homogeneity of the data and inevitably influencing the choice of patients. Secondly, the SEER database only provides general information about the patients and the relevant treatment modalities, and does not provide information about the patients’ specific treatments such as chemotherapy and radiotherapy, which may lead to a certain degree of bias in the study results. Finally, although our study showed that the constructed model had positive predictive value, external validation in prospective clinical trials is still needed. Despite these limitations, we have determined the epidemiological characteristics, independent prognostic factors, and information on OS and DSS of patients with HIV-associated CTCL.

## Conclusions

Our study shows that HIV-associated CTCL is very rare, and the incidence has been on the rise in the last decade or so. We determined the demographic, clinical characteristics and survival-related prognostic factors of patients with HIV-associated CTCL, and constructed and validated nomograms of OS, DSS of patients at different time points. The nomograms can help clinicians to better identify the individual risks of patients and accurately assess the prognosis of patients.

## Data Availability Statement

The original contributions presented in the study are included in the article/**Supplementary Material**. Further inquiries can be directed to the corresponding authors.

## Author Contributions

ZY, YS, and QH conceived the study idea, and performed interpretation, manuscript writing, and final approval. DG and FH performed the data analysis and collection, as well as language polishing. All authors reviewed and approved the final version of the manuscript.

## Funding

This work was supported by Hubei Province Health and Family Planning Scientific Research Project (WJ2021M261 to YS) and the Natural Science Foundation of Hubei Province (2019CFB567 to YS).

## Conflict of Interest

The authors declare that the research was conducted in the absence of any commercial or financial relationships that could be construed as a potential conflict of interest.

## Publisher’s Note

All claims expressed in this article are solely those of the authors and do not necessarily represent those of their affiliated organizations, or those of the publisher, the editors and the reviewers. Any product that may be evaluated in this article, or claim that may be made by its manufacturer, is not guaranteed or endorsed by the publisher.
