# Supplementary material for: Epidemiological Characteristics and the Development of Prognostic Nomograms of Patients With HIV-Associated Cutaneous T-Cell Lymphoma
Source: Front Oncol. 2022 Mar 15;12:847710. doi: 10.3389/fonc.2022.847710 (PMC8965059; doi:10.3389/fonc.2022.847710)
Supplement: Supplementary file 1 [file Table_1.docx]

**Supplementary Table 1** Incidence and APC of male and female HIV-associated CTCL patients by year.

| Cohort | Segment | Lower Endpoint | Upper Endpoint | APC | Lower CI | Upper CI | Test Statistic (t) | Prob > \|t\| |
| --- | --- | --- | --- | --- | --- | --- | --- | --- |
| Male and female | 1 | 2004 | 2007 | -14 | -65.3 | 113.4 | -0.4 | 0.699 |
| Male and female | 2 | 2007 | 2011 | 100.6* | 19.9 | 235.5 | 3.3 | 0.016 |
| Male and female | 3 | 2011 | 2017 | 10.2* | 3.2 | 17.7 | 3.6 | 0.011 |
| Male | 1 | 2004 | 2013 | 41.7* | 23.7 | 62.3 | 5.8 | < 0.001 |
| Male | 2 | 2013 | 2017 | 3.7 | -12.1 | 22.3 | 0.5 | 0.634 |
| Female | 1 | 2004 | 2012 | 57.1* | 37.1 | 80.1 | 7.5 | < 0.001 |
| Female | 2 | 2012 | 2017 | 8.3 | -0.8 | 18.3 | 2.1 | 0.07 |

APC, annual percentage change; HIV-associated CTCL, human immunodeficiency virus associated cutaneous T-Cell lymphoma; CI, confidence intervals;

* *P* < 0.05 was considered statistically significant.
