# Supplementary material for: Epidemiological Characteristics and the Development of Prognostic Nomograms of Patients With HIV-Associated Cutaneous T-Cell Lymphoma
Source: Front Oncol. 2022 Mar 15;12:847710. doi: 10.3389/fonc.2022.847710 (PMC8965059; doi:10.3389/fonc.2022.847710)
Supplement: Supplementary file 2 [file Table_2.docx]

**Supplementary Table 2** Population of male and female HIV-associated CTCL patients by year.

| Cohort | Year of diagnosis | Observed Population | Standard Error | Modeled Population | Joinpoint Location |
| --- | --- | --- | --- | --- | --- |
| Male and female | 2004 | 0.01 | 0 | 0.01 |  |
| Male and female | 2005 | 0.01 | 0 | 0.01 |  |
| Male and female | 2006 | 0.01 | 0 | 0.01 |  |
| Male and female | 2007 | 0.01 | 0 | 0.01 | Joinpoint 1 |
| Male and female | 2008 | 0.01 | 0 | 0.01 |  |
| Male and female | 2009 | 0.03 | 0.01 | 0.02 |  |
| Male and female | 2010 | 0.04 | 0.01 | 0.05 |  |
| Male and female | 2011 | 0.09 | 0.01 | 0.09 | Joinpoint 2 |
| Male and female | 2012 | 0.09 | 0.01 | 0.1 |  |
| Male and female | 2013 | 0.13 | 0.01 | 0.11 |  |
| Male and female | 2014 | 0.14 | 0.01 | 0.12 |  |
| Male and female | 2015 | 0.13 | 0.01 | 0.14 |  |
| Male and female | 2016 | 0.13 | 0.01 | 0.15 |  |
| Male and female | 2017 | 0.17 | 0.01 | 0.17 |  |
| Male | 2004 | 0.01 | 0.01 | 0.01 |  |
| Male | 2005 | 0.02 | 0.01 | 0.01 |  |
| Male | 2006 | 0.01 | 0 | 0.01 |  |
| Male | 2007 | 0.01 | 0 | 0.02 |  |
| Male | 2008 | 0.02 | 0.01 | 0.03 |  |
| Male | 2009 | 0.03 | 0.01 | 0.04 |  |
| Male | 2010 | 0.04 | 0.01 | 0.06 |  |
| Male | 2011 | 0.12 | 0.02 | 0.08 |  |
| Male | 2012 | 0.12 | 0.02 | 0.12 |  |
| Male | 2013 | 0.17 | 0.02 | 0.17 | Joinpoint 1 |
| Male | 2014 | 0.18 | 0.02 | 0.17 |  |
| Male | 2015 | 0.16 | 0.02 | 0.18 |  |
| Male | 2016 | 0.15 | 0.02 | 0.19 |  |
| Male | 2017 | 0.22 | 0.02 | 0.19 |  |
| Female | 2004 | 0 | 0 | 0 |  |
| Female | 2005 | 0 | 0 | 0 |  |
| Female | 2006 | 0 | 0 | 0.01 |  |
| Female | 2007 | 0.01 | 0 | 0.01 |  |
| Female | 2008 | 0 | 0 | 0.01 |  |
| Female | 2009 | 0.02 | 0.01 | 0.02 |  |
| Female | 2010 | 0.03 | 0.01 | 0.03 |  |
| Female | 2011 | 0.06 | 0.01 | 0.05 |  |
| Female | 2012 | 0.08 | 0.01 | 0.08 | Joinpoint 1 |
| Female | 2013 | 0.09 | 0.01 | 0.09 |  |
| Female | 2014 | 0.11 | 0.02 | 0.1 |  |
| Female | 2015 | 0.1 | 0.01 | 0.11 |  |
| Female | 2016 | 0.12 | 0.02 | 0.12 |  |
| Female | 2017 | 0.13 | 0.02 | 0.13 |  |

HIV-associated CTCL, human immunodeficiency virus associated cutaneous T-Cell lymphoma.
